# Endocrinology Clinical Skills Assessment Program: A Structured Clinical Assessment Program to Enhance Clinical Reasoning in Subspecialty Training

**DOI:** 10.7759/cureus.93834

**Published:** 2025-10-04

**Authors:** Hima Darapu, Chinenye O Usoh

**Affiliations:** 1 Internal Medicine/Endocrinology, Wake Forest University School of Medicine, Winston-Salem, USA; 2 Endocrinology, Diabetes and Metabolism, Veterans Affairs Medical Center, Salisbury, USA

**Keywords:** case-based learning, clinical reasoning, clinical skills assessment, endocrinology fellows, medical education, structured assessment

## Abstract

Introduction: The Endocrinology Clinical Skills Assessment Program (ECSAP) was developed at Wake Forest University School of Medicine to enhance history-taking, diagnostic reasoning, and personalized feedback in endocrinology fellows. Recognizing a gap in formative clinical skill evaluation, ECSAP introduces standardized case-based assessments across key endocrine topics.

Methods: ECSAP sessions occur twice yearly and include four 25-minute standardized clinical scenarios (e.g., adrenal incidentaloma, hypercalcemia). Each case is pre-assigned to faculty, who provide fellows with a one-line prompt and standardized responses. Fellows navigate history, physical exam, labs/imaging, and assessment/plan components in real time. Faculty provide structured feedback and score fellows using detailed rubrics. Evaluations from both fellows and faculty assess program effectiveness.

Results: Over one academic year, ECSAP was implemented in two separate sessions, each involving four fellows and four to six faculty members. Despite the small cohort size, the consistency of responses across both sessions reinforces the reliability and perceived value of the program. Fellow evaluations demonstrated strong endorsement, with 100% agreeing that ECSAP improved diagnostic reasoning and helped identify knowledge gaps, and an average feedback usefulness rating of 4.8 out of 5. Faculty evaluations similarly reflected high satisfaction, with 100% reporting that ECSAP enhanced their ability to assess clinical skills and provide actionable feedback, yielding an average rating of 4.7 out of 5. These reproducible, positive outcomes across repeated implementations support the feasibility and educational impact of ECSAP as a complementary structured formative assessment model.

Conclusion: ECSAP is a reproducible, low-resource, case-based formative assessment model that complements existing clinical training. Fellows benefit from real-time feedback, while faculty gain structured opportunities for formative evaluation. Future directions include integration with competency-based milestones and expansion to other subspecialties.

## Introduction

Competency-based medical education emphasizes not only knowledge acquisition but also the development of clinical reasoning, communication skills, and diagnostic acumen in real-world scenarios [1]. While traditional evaluations in endocrinology fellowships rely heavily on longitudinal clinical performance assessments, direct observation and structured feedback are often limited due to clinical time constraints, variable patient encounters, and inconsistency in evaluator engagement [2,3]. These limitations can result in gaps in formative feedback and hinder the early identification of specific areas for skill development. Core characteristics of effective feedback include timely, frequent, criterion-based, use of multiple data sources, reciprocal, and involving skillful interaction [4]. In a busy clinical environment, both learners and teachers have noted difficulty balancing patient care with timely feedback, in addition to variability in feedback style as barriers to achieving effective feedback [5]. These challenges were further amplified during the COVID-19 pandemic, which disrupted clinical routines and reduced opportunities for in-person supervision and feedback. A systematic review from the United Kingdom during this time reported changes to postgraduate education as having a significant negative impact on trainee progression [6]. Recognizing the need for a more adaptable approach to clinical skills assessment, we developed the Endocrinology Clinical Skills Assessment Program (ECSAP) at Wake Forest University School of Medicine. ECSAP was designed as a faculty-facilitated, case-based clinical simulation model that allows fellows to demonstrate and refine their diagnostic reasoning and clinical communication skills in a time-controlled, low-stakes environment. It incorporates elements of standardized assessment, formative evaluation, and immediate feedback, aligning with best practices in adult learning theory and competency-based medical education.

Our intention with ECSAP was threefold: (1) To create structured opportunities for fellows to practice and be assessed on essential clinical skills, including history-taking, physical exam, data synthesis, clinical reasoning, and management planning within a standardized and reproducible format. This approach ensures deliberate practice of concepts that are often overlooked or not explicitly addressed during busy clinical rotations, yet remain clinically relevant; (2) To enable faculty to directly observe fellow performance using standardized clinical scenarios, thereby minimizing variability in assessment and ensuring a more equitable and consistent evaluation process across all learners; (3) To foster a culture of feedback and reflection, providing fellows with personalized, actionable insights that can be immediately applied to clinical practice. These formative interactions are designed to reinforce strengths, identify knowledge gaps, and support continuous professional growth.

Each ECSAP session simulates four common endocrinology presentations, such as adrenal incidentaloma, hypercalcemia, pituitary incidentaloma, or abnormal thyroid function. Fellows are evaluated using detailed scoring rubrics created by faculty content experts. These rubrics emphasize the completeness and relevance of clinical reasoning, rather than simply reaching the correct diagnosis. At the conclusion of each case, faculty provide real-time feedback and highlight both strengths and areas for improvement. By intentionally separating this exercise from routine clinical care and standard evaluations, ECSAP offers a reproducible, focused framework to assess and improve fellows' readiness for independent endocrine practice. This manuscript describes the structure, implementation, and outcomes of ECSAP and proposes its utility as a model for clinical assessment in other subspecialties.

The objective of this study was to evaluate the ECSAP program using feedback from endocrine fellows and faculty preceptors. Specifically, we aimed to demonstrate that the program addresses time constraints commonly encountered in clinical settings, facilitates real-time self-assessment by fellows and faculty identification of knowledge gaps, and promotes equitable evaluation by standardizing case assignments across faculty.

## Materials and methods

Program design

The ECSAP is conducted semiannually, with sessions scheduled in both the Fall and Spring semesters. Each session lasts approximately two hours and is designed to accommodate all participating fellows in a structured, time-efficient manner. The program was intentionally designed to replicate real-world clinical encounters while allowing for direct observation, structured feedback, and formative evaluation. ECSAP sessions were scheduled during the regular didactic time block, requiring no additional time commitment from the faculty or fellows.

Structure and Flow

Each fellow participates in four consecutive one-on-one clinical encounters, each lasting approximately 20-25 minutes, with different faculty facilitators. All faculty members work from standardized case prompts and scoring rubrics to ensure consistency across encounters. Each session includes four distinct endocrine cases. Each case is assigned to a specific faculty member who delivers the same case to all fellows, ensuring equity in assessment and standardization of scoring. Fellows rotate through all four cases sequentially.

Each 25-minute case is broken down into the following components:

History-taking (10 minutes): Fellows are presented with a one-line clinical prompt (e.g., “35-year-old with adrenal incidentaloma”) and are expected to lead a focused and thorough clinical interview. Faculty provide pre-scripted responses based on the fellow’s line of questioning. Fellows are evaluated on the relevance and depth of their questioning.

Physical examination and diagnostic data review (7 minutes): Fellows ask targeted questions regarding physical exam findings and request relevant laboratory or imaging data. Faculty provide information based on standardized responses aligned with the case rubric. This segment tests the fellow’s ability to prioritize key findings and interpret available information.

Assessment and plan (5 minutes): Fellows synthesize the information gathered to develop a differential diagnosis, formulate a working diagnosis, and present a concise management plan. This segment emphasizes the fellow’s clinical reasoning, integration of findings, and decision-making.

Concept-based knowledge questions (integrated throughout): During the encounter, faculty may pose brief, targeted questions related to clinical concepts, physiology, or diagnostic reasoning specific to the case. These questions are used to assess the depth of understanding and reinforce high-yield teaching points.

Feedback and teaching (5 minutes): Faculty conclude the session by providing immediate, individualized feedback. Emphasis is placed on clinical reasoning, communication skills, and completeness of the evaluation. Teaching pearls relevant to the case are shared, and areas for improvement are highlighted to promote reflective learning.

Case Development

Faculty members collaboratively designed standardized clinical cases representing high-yield endocrine conditions commonly encountered in fellowship training, including adrenal incidentaloma, pituitary incidentaloma, abnormal thyroid function tests, and hypercalcemia. Each case is carefully structured to promote consistency, fairness, and educational value across all fellow encounters. Each case includes the following components: one-line introductory prompt, pre-scripted faculty responses, concept-based testing questions, and a detailed scoring rubric. The scoring rubric is a comprehensive evaluation (total score range: 70-100 points), allowing faculty to assess performance across key domains such as history-taking, physical exam findings, data synthesis, clinical reasoning, and management planning. Rubrics are provided in Appendices A-C, and additional case-specific examples are available upon request. Points were awarded based on the fellow’s ability to address key elements within each domain, reflecting both the thoroughness and accuracy of their history-taking. Further scoring for the final assessment/plan was based on the fellow’s ability to articulate sound clinical reasoning that supported their ultimate management approach.

Faculty Role As Facilitators

Faculty members play a central role in the success of ECSAP, serving as educational facilitators, coaches, and observers. Their responsibilities extend throughout the planning, implementation, and feedback phases of the program to ensure a standardized, equitable, and educational experience for all fellows. To promote scoring consistency and minimize bias, the same faculty member presented each case to all fellows, and faculty preceptors participated in an educational session on bias reduction prior to ECSAP implementation. This approach supported standardization and reliability across assessments.

Real-Time Facilitation and Observation

During each 25-minute encounter, faculty are responsible for introducing the case prompt to the fellow without additional explanation; providing only pre-scripted responses to fellow-initiated questions related to history, physical exam findings, laboratory data, and imaging, ensuring consistent information delivery across learners; maintaining neutrality, refraining from coaching, leading, or offering unsolicited prompts that may influence the fellow’s clinical reasoning or decision-making; posing concept-based questions at designated points to assess clinical knowledge and reasoning; monitoring time, providing subtle cues to help fellows transition between sections (e.g., from history to exam); delivering personalized, actionable feedback during the final segment of the session, focusing on clinical reasoning, communication, and knowledge gaps identified during the encounter; completing and submitting case-specific scoring rubrics (see Appendices A-C) for each fellow.

Fellow Role and Expectations

Fellows serve as active participants in ECSAP, engaging in structured, time-limited clinical scenarios that simulate real-world endocrine consultations. They are expected to demonstrate clinical reasoning, diagnostic accuracy, and communication skills while approaching the program as a formative learning experience that promotes reflective practice and professional growth. Each fellow participates in four standardized clinical scenarios per session, rotating through one-on-one encounters with faculty facilitators. Their responsibilities include:

Engaging in structured clinical reasoning: Fellows begin each case with a one-line prompt from faculty and are expected to lead a comprehensive evaluation by asking focused questions, requesting relevant physical exam findings, ordering appropriate labs and imaging, and synthesizing clinical data to formulate an assessment and plan.

Demonstrating clinical knowledge and decision-making: Fellows are expected to apply their understanding of endocrine physiology, diagnostics, and management strategies. Faculty may intersperse concept-based questions throughout the case to assess depth of understanding and promote active learning.

Managing time effectively: Fellows are responsible for navigating each case within a 25-minute time frame, balancing thorough inquiry with efficient decision-making. They are encouraged to move purposefully through history-taking, diagnostics, and clinical reasoning to ensure adequate time for feedback.

Receiving and applying feedback: At the conclusion of each case, fellows receive direct, individualized feedback from faculty regarding clinical strengths, areas for improvement, and key teaching points. Fellows are expected to engage in this feedback process with openness and use it to inform ongoing skill development.

Evaluation tools

Two structured evaluation forms were used to assess ECSAP’s effectiveness and guide future improvements. These tools provide valuable insight into both learner outcomes and faculty experience and support iterative refinement of the program.

Fellow Evaluation Form

Completed anonymously post-session, this form uses Likert scales and open-ended questions to assess perceived improvements in clinical skills, usefulness of feedback, session structure, and suggestions for future topics (see Appendices A-C).

Faculty Evaluation Form

Completed after all sessions, this form captures faculty perspectives on the program’s educational value, fairness, effectiveness in assessing fellows, and logistical considerations (see Appendices A-C).

## Results

Four fellows and six faculty participated in ECSAP over two academic cycles. Immediately after each session, participants were asked to complete an evaluation of the ECSAP program. Questions assessing the effectiveness of the program in meeting its educational objectives were assessed using a 5-point Likert scale (1 = strongly agree, 5 = strongly disagree). The questionnaire was voluntary and distributed as a paper survey.

Fellows who participated either strongly agreed (66.7%) or agreed (33.3%) that ECSAP enhanced history-taking skills and improved diagnostic reasoning. Most (66.7%) strongly agreed that the structured feedback helped identify gaps not addressed in routine clinics. One hundred percent of fellows strongly agreed that ECSAP covered minor details in history-taking and assessment that are often missed in clinical settings (Table 1).

**Table 1:** Fellow Evaluation of ECSAP Program Objectives List of ECSAP educational objectives and participant feedback (from fellow evaluation form) on whether stated goals were achieved. Evaluation forms were completed anonymously post-session, using Likert scales and open-ended questions to assess perceived improvements in clinical skills, usefulness of feedback, and session structure. ECSAP: Endocrinology Clinical Skills Assessment Program

Item (n=6)	Strongly Agree	Agree	Neutral	Disagree	Strongly Disagree
ECSAP met its stated goals in the following areas:					
Enhance history-taking skills	66.7%	33.3%	-	-	-
Improve diagnostic reasoning and enhance the ability to form assessments and plans	66.7%	33.3%	-	-	-
Cover minor details in history-taking that are often missed in clinical scenarios	100%	-	-	-	-
Receive clear, real-time feedback from facilitators	100%	-	-	-	-
Help identify and address knowledge gaps	66.7%	33.3%	-	-	-

When asked to rate the overall educational value on a scale of 1-5 (1 being lowest and 5 being highest), the average response was 4.8± 0.40. Overall, ECSAP was well received by fellows who also provided positive comments (Table 2).

**Table 2:** Fellow and Faculty Narrative Comments Narrative comments from fellow and faculty participants. Evaluation forms were completed anonymously post-session, using Likert scales and open-ended questions to assess the program’s benefits and also potential areas for improvement. ECSAP: Endocrinology Clinical Skills Assessment Program

Question		Quotations
	Fellows	Faculty
Would you recommend any new cases or topics for future ECSAP sessions?	Thought cases were clinically appropriate and helpful	It might be interesting to use cases that correlate with topics that the fellows have struggled with on the in-service exam.
	More adrenal, thyroid, and pituitary topics helpful	Hypoglycemia and hyperthyroidism
	Include more adrenal and tumors	Polyuria/Diabetes insipidus, choosing diabetes medication regimen (non-insulin)
	Enjoyed the variety across the two sessions we’ve had so far	-
What aspect of ECSAP did you find most beneficial?	I really liked the prompted questions	Identifying knowledge gaps in fellows and determining how confident they are in managing real-life cases
	Working through a case from scratch without the electronic health record and then getting immediate feedback	Ability for real-time feedback to the fellow
	Real-time feedback and doing cases 1:1 with attending	The time to have a real in-depth conversation about a patient whom we don’t have the time for in actual clinic.
	Feedback and minor history details	-

Faculty facilitators also gave positive feedback on the ECSAP program (Table 2). All either agreed or strongly agreed that the program helped faculty provide specific actionable insights to improve the fellow’s clinical skills. When asked to rate the overall educational value on a scale of 1-5 (1 being lowest and 5 being highest), the average response was 4.7± 0.49. No major concerns about bias or fairness were identified. The most beneficial aspects cited included the opportunity to provide real-time feedback and identify knowledge gaps in the fellow.

## Discussion

ECSAP was designed to address a recognized gap in structured, formative assessment during subspecialty fellowship training. Traditional evaluations often rely on ad hoc clinical observations and written assessments, which may lack standardization, direct observation, or immediate feedback [7]. ECSAP introduced a reproducible, low-resource model that complements these traditional approaches by focusing on deliberate practice, real-time feedback, and competency development.

Similar structured assessments, such as Objective Structured Clinical Examinations (OSCEs), have been widely implemented in undergraduate and some residency training settings to improve clinical reasoning and feedback quality [8-10]. Our findings align with previous reviews highlighting that simulation-based assessments, particularly those using structured, repeatable formats, can provide valid and reliable evaluation of clinical reasoning and technical skills [11]. Like ECSAP, successful models often incorporate multiple cases, standardized scoring rubrics, and real-time feedback, reinforcing their utility in formative assessment. However, as noted in Ryall et al.’s review, simulation assessments should ideally complement, rather than replace, traditional evaluation methods to ensure comprehensive competency evaluation [11].

The Mini-Clinical Evaluation Exercise (Mini-CEX) is a widely used formative tool for assessing clinical skills during real patient encounters, including history-taking, physical exam, and clinical judgment. However, its effectiveness may be limited by variability in case complexity and evaluator subjectivity [12]. In contrast, ECSAP provides a standardized, reproducible format with scripted cases and structured rubrics, promoting consistent assessment and feedback. By controlling patient variability, ECSAP ensures greater equity and overcomes limitations seen in traditional evaluations. There have been multiple studies showing the impact of harmful bias in medical education assessments [13,14]. Social identity characteristics (SIDCs) such as gender, race, religious expression, age, and ethnicity are inconsistently studied [15]. Recommendations to overcome evaluator bias have included creating assessments that foster learning and improved outcomes, promoting continuous quality improvement of the evaluation, and providing fair learning and assessment environments [16]. The ECSAP aimed to curb assessment bias by using standardized clinical cases to evaluate learners. ECSAP also provided space for learner and evaluator feedback to allow for assessment improvement.

Our results demonstrate that ECSAP was well received by both fellows and faculty. Fellows reported significant improvement in their diagnostic reasoning, history-taking, and synthesis of clinical information. The structure of ECSAP allowed them to deliberately practice skills in a focused and low-pressure environment, which many identified as highly educational compared to typical inpatient or outpatient workflow. Faculty valued ECSAP for its ability to uncover subtle strengths and deficits in fellow performance that may otherwise go unrecognized in the traditional clinical setting. Moreover, the structured scoring rubric and consistent case delivery helped reduce bias and encouraged equitable assessment across learners. ECSAP’s design incorporates several deliberate strategies to enhance educational value while minimizing bias and ensuring fairness in assessment. Faculty feedback confirmed that this structure enabled objective, reproducible, and unbiased assessment, while providing a fair and supportive environment for fellows to demonstrate their clinical skills. The integration of concept-based knowledge questions throughout the cases further reinforced key physiologic and diagnostic principles, encouraging deeper cognitive engagement and critical thinking.

ECSAP was intended to complement, not replace, existing assessment strategies, offering a structured and standardized format for skills evaluation alongside traditional methods. ECSAP was intentionally designed to be low-cost and logistically feasible. Sessions were held in faculty offices using printed rubrics and minimal infrastructure. Faculty facilitators did not require specialized simulation training, and the cases were built around high-yield, real-world endocrine problems. This simplicity makes ECSAP easily adaptable to other fellowship programs or subspecialty divisions seeking to implement structured formative assessments without the need for simulation centers or additional administrative resources. Faculty feedback suggested that short pre-session calibration discussions could enhance consistency in scoring and feedback delivery across facilitators. Some also recommended expanding the variety and complexity of cases in future iterations, including integration of communication-focused scenarios (e.g., delivering bad news, navigating diagnostic uncertainty).

Future directions of the program

ECSAP is a dynamic initiative that will continue to evolve in response to ongoing feedback from both fellows and faculty. Planned enhancements aim to strengthen its educational impact, broaden its scope, and improve scalability and integration with broader training objectives. Key future directions include:

Expansion of the Case Library

To ensure broader clinical coverage and increase relevance, additional cases are being developed in areas such as thyroid cancer evaluation, complex diabetes management scenarios, and reproductive endocrinology. These topics reflect high-impact clinical conditions that fellows may not consistently encounter during routine rotations but are essential for comprehensive endocrine training.

Digitalization of Evaluation Tools

Plans are underway to transition from paper-based scoring to electronic assessment platforms. This will allow for more efficient data collection, streamlined feedback, centralized performance tracking, and potential integration with program evaluation dashboards.

Alignment With Accreditation Council for Graduate Medical Education (ACGME) Milestones

ECSAP scoring rubrics will be refined to map directly to ACGME sub-competencies and milestones, enabling program leadership to use ECSAP as a formal competency assessment tool. This alignment will support more robust documentation of fellow progression and readiness for independent practice.

Longitudinal Performance Tracking

ECSAP can facilitate longitudinal analysis of individual fellow performance across sessions. Trends over time will help identify areas of improvement or concern, guide personalized educational plans, and provide additional data for Clinical Competency Committees (CCCs).

Scalability Across Subspecialties

Given its low-resource requirements, reproducible format, and emphasis on structured faculty-fellow interaction, ECSAP shows promise as a scalable model for other subspecialty training programs. It provides a structured method to assess clinical reasoning and decision-making without relying on real-life patient encounters, making it especially useful in outpatient specialties or settings with limited direct observation opportunities. With its foundation in real-time interaction, immediate feedback, and reflective learning, ECSAP holds significant promise as a template for competency-based assessment in graduate medical education, offering a practical bridge between educational theory and clinical training. Additional validation in larger, multi-center studies is needed to further assess.

Limitations of the study

While ECSAP has demonstrated educational value and feasibility, a few limitations must be acknowledged. Although cases are based on real scenarios, the absence of standardized patients or live role-play reduces realism and limits assessment of interpersonal communication, empathy, patient education, and physical examination skills. The 25-minute case structure may limit the depth of clinical exploration and reduce time for comprehensive feedback, particularly for complex or nuanced topics. Despite standardized rubrics and scripted responses, variability in faculty delivery and judgment may have introduced variability in scoring. Inter-rater reliability has not yet been assessed, and formal calibration has not been implemented. ECSAP has thus far been implemented within a single endocrinology fellowship program with a small number of participants. The small sample size and single institution setting limit the ability to draw definitive conclusions, although findings were supported by repeated assessments. Additional research across multiple sites and larger cohorts is necessary to validate the ECSAP model. Regardless, ECSAP’s structured case-based format, minimal resource requirements, and emphasis on faculty-led feedback make it a highly adaptable model that can be readily implemented across other subspecialties and training institutions. The program also relies on dedicated faculty time and participation, which may pose challenges for scalability in settings with limited faculty availability.

## Conclusions

ECSAP represents a practical, structured, and learner-centered approach to clinical skills assessment in endocrinology fellowship training. By combining standardized case-based scenarios with real-time faculty observation and feedback, the program addresses a critical gap in formative evaluation that often exists in busy clinical settings. Fellows benefit from targeted opportunities to refine diagnostic reasoning, synthesize clinical data, and receive actionable feedback, while faculty gain a consistent and equitable framework to assess and support learner growth. The program’s simplicity, scalability, and emphasis on core educational principles, deliberate practice, formative assessment, and reflective learning make ECSAP a valuable model not only for endocrine training but also for other subspecialty programs seeking to enhance competency-based education. ECSAP should be viewed as an adjunct to traditional assessment methods, providing structured opportunities for evaluating clinical reasoning and decision-making, while recognizing that additional tools are needed to assess domains such as physical examination. As ECSAP continues to evolve with digital integration, milestone alignment, and longitudinal tracking, it holds strong potential to contribute meaningfully to the advancement of structured clinical assessment in graduate medical education.

## Figures and Tables

**Figure 1:**
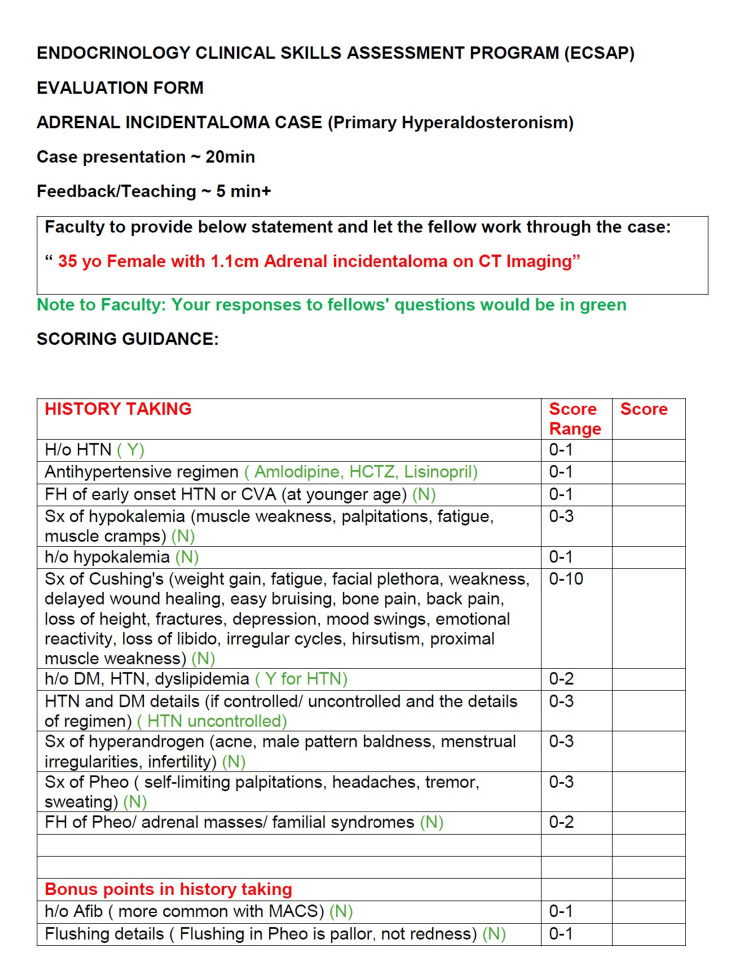
ECSAP Evaluation Form (Sample, Page 1) ECSAP: Endocrinology Clinical Skills Assessment Program

**Figure 2:**
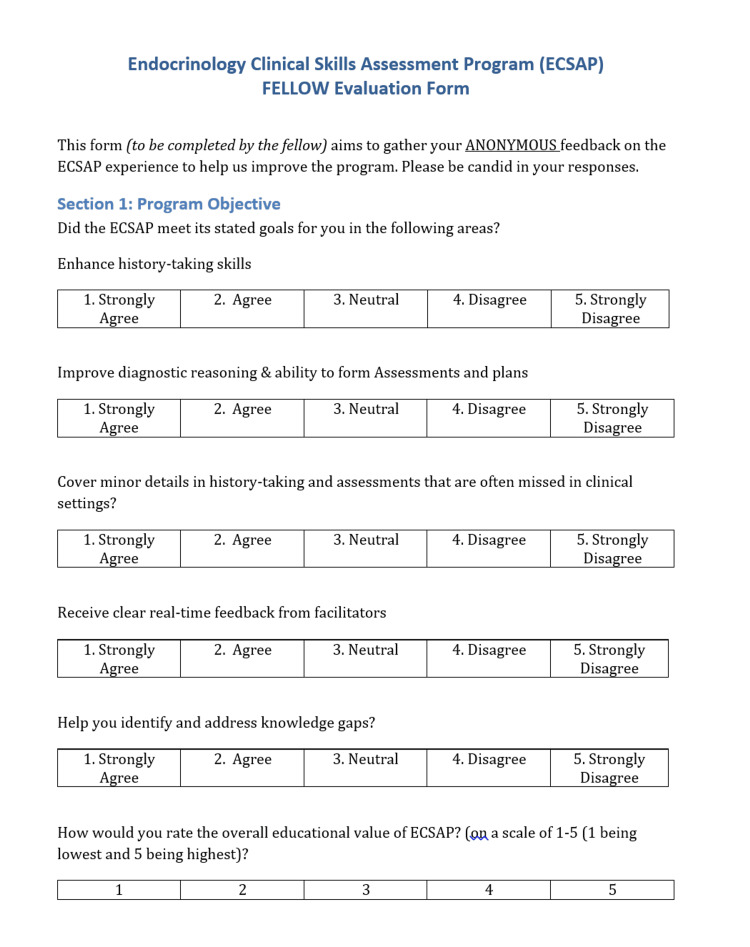
ECSAP Fellow Evaluation Form (Page 1) ECSAP: Endocrinology Clinical Skills Assessment Program

**Figure 3:**
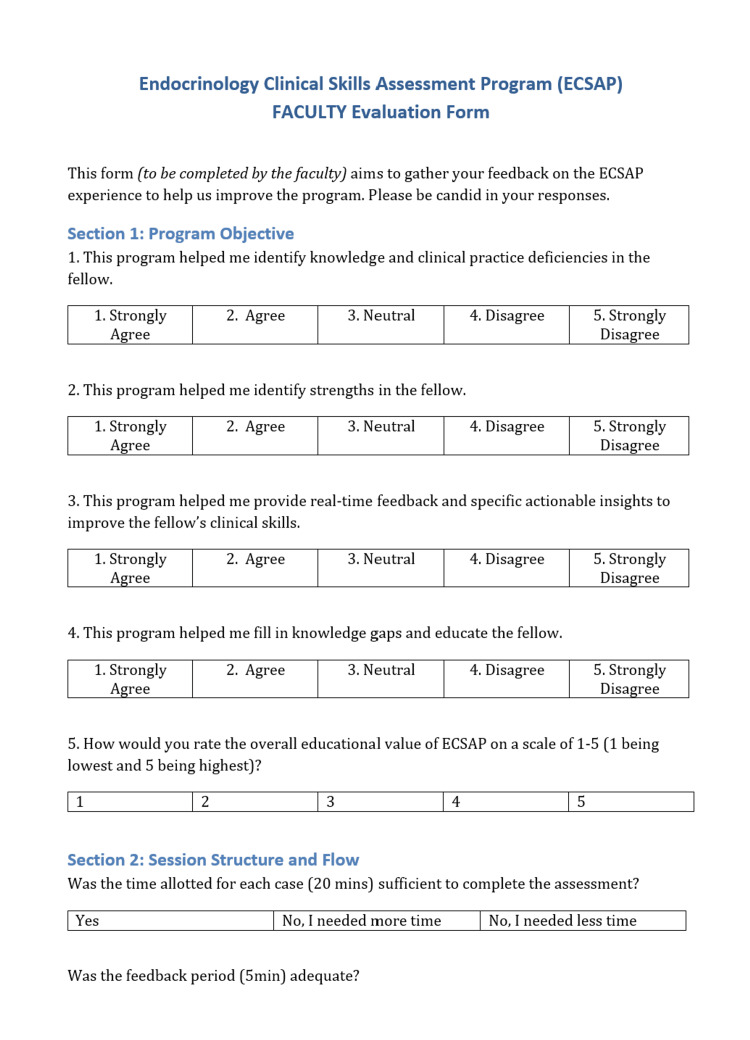
ECSAP Faculty Evaluation Form (Sample, Page 1) ECSAP: Endocrinology Clinical Skills Assessment Program

## Appendices

Appendix A 

Appendix B 

Appendix C 
